# Elevated CO_2_ and the hidden hunger crisis: mechanisms, magnitudes, and mitigation of micronutrient dilution in staple crops

**DOI:** 10.3389/fpls.2026.1929375

**Published:** 2026-09-16

**Authors:** Saravanan Raju, Anusree Kookal, Manju R.V., Susan Joshy, Musti Sree Phani Bhavya, Amrutha E.A., Jani Aushna, Neethu Chandra C.

**Affiliations:** 1 Division of Crop Production, ICAR-Central Tuber Crops Research Institute, Thiruvananthapuram, India; 2 Department of Plant Physiology, College of Agricutlture Vellayani, Thiruvananthapuram, India

**Keywords:** elevated CO_2_, face, hidden hunger, iron, micronutrient dilution, zinc

## Abstract

Hidden hunger results from inadequate intake of iron (Fe), zinc (Zn), and other key micronutrients. Over 2 billion people worldwide are affected, especially those who rely on staple cereals for daily food. Elevated CO_2_ often boosts photosynthesis and yield in C_3_ crops. However, it also reduces grain mineral levels via multiple physiological pathways. These include less transpiration-driven mineral delivery, an imbalance between carbon accumulation and mineral uptake, and changes in mineral uptake, transport, and grain loading. This review collects current evidence for Fe, Zn, copper (Cu), manganese (Mn), boron (B), molybdenum (Mo), selenium (Se), and iodine (I). It draws on free-air CO_2_ enrichment (FACE) experiments, controlled studies, reviews, and meta-analyses from major cereals and legumes. Grain Fe and Zn show the most consistent and significant declines, and often drop more under combined CO_2_ enrichment with drought, warming, or low nitrogen. Cu and Mn show greater variability, and data for Se, I, B, and Mo remain limited. This highlights big gaps in climate–nutrition research. The review also considers other factors that affect micronutrient dilution. It identifies research priorities and assesses strategies such as breeding, biofortification, agronomic management, soil–microbiome support, fortification, and dietary diversity. Overall, the evidence indicates that rising CO_2_ levels threaten the nutritional quality of grains. There is a clear need to include micronutrient density in climate-resilient crop improvement and food system planning.

## Graphical Abstract

**Figure:**
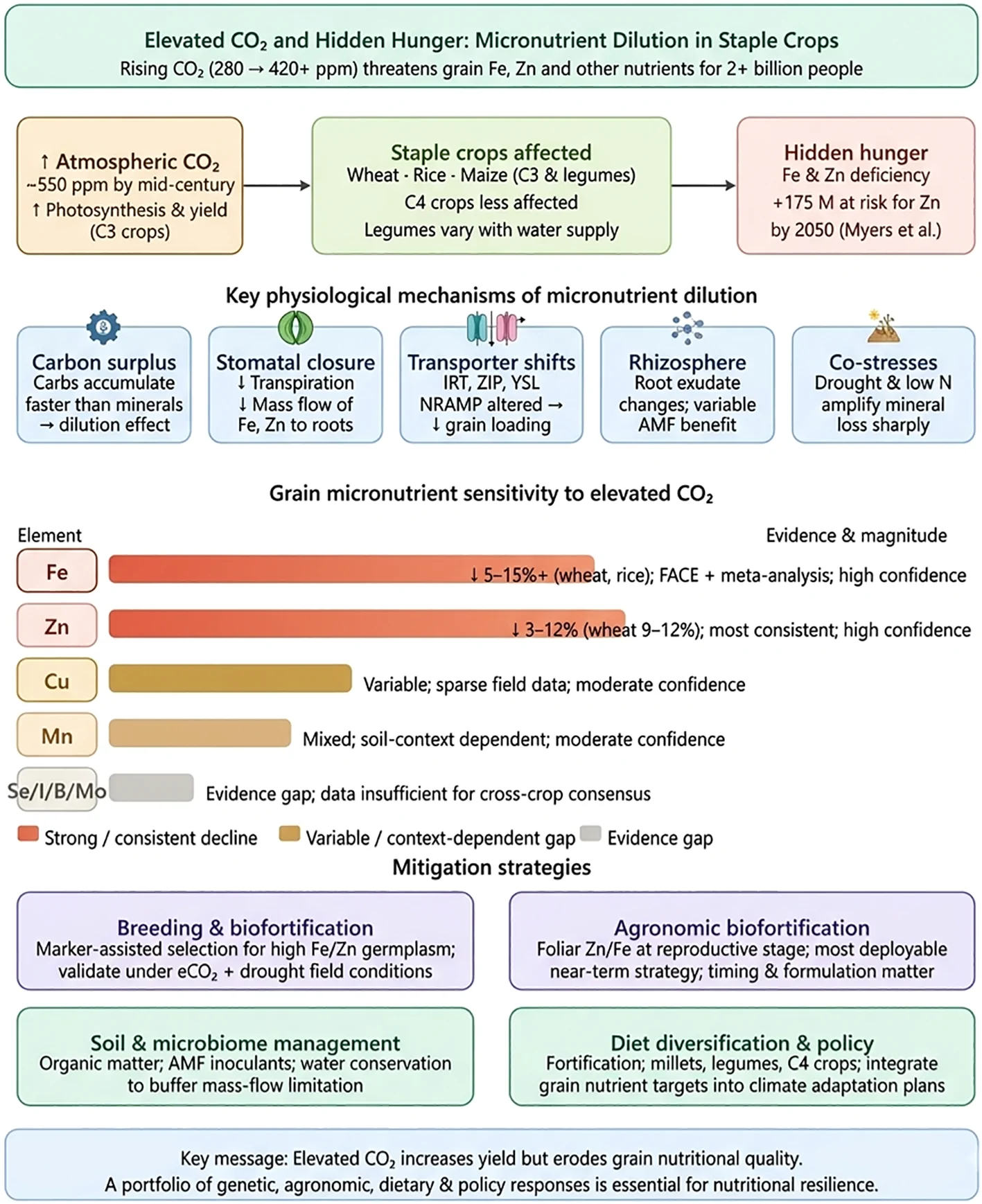

## 1 Introduction

Over two billion people worldwide lack essential vitamins and minerals, a condition known as hidden hunger (Gödecke et al., 2018). It develops quietly, usually without obvious signs of energy deficiency (Lowe, 2021). Iron (Fe) and zinc (Zn) deficiencies are especially important: zinc deficiency weakens cognitive and motor development in children (Black, 2003), while iron deficiency reduces physical work capacity (Haas and Brownlie, 2001), and both compromise immunity and maternal and child health. These problems are worst in low- and middle-income countries with limited diet variety, where the burden is tied to cereal-based diets (Gödecke et al., 2018). Wheat, rice, and maize give much of the daily energy for at-risk groups, yet these staples usually offer only modest levels of micronutrients the body can use. Any drop in grain Fe or Zn concentration can worsen nutritional vulnerability, and this can occur even when people consume enough calories (Lowe, 2021).

Rising atmospheric CO_2_ is one such threat SSP2-4.5 projected to reach 550 ppm by 2050 (Riahi et al., 2022) is now seen as a major threat to crop nutrition and human micronutrient security. CO_2_ has increased from about 280 ppm before industrialisation to 420.0 ppm in 2023, 151% of the pre-industrial level (Lindsey and Dahlman, 2020). Elevated CO_2_ (eCO_2_) usually increases photosynthesis and biomass in C_3_ crops, but this yield advantage often comes at the cost of lower grain mineral levels (Myers et al., 2014). Over 30 years, FACE studies have found that C_3_ crop yields rose by about 18%, while seed Fe and Zn often fell under eCO_2_ (Ainsworth and Long, 2021). In rice, a systematic review found that eCO_2_ often reduces grain Fe and Zn, with many studies showing losses of over 10% at mid-century CO_2_ levels (Chumley and Hewlings, 2020). One meta-analysis with 542 observations across major crops also showed significant drops in grain Zn and Fe under eCO_2_, with these effects worse under low nitrogen or drought conditions (Al-Hadeethi et al., 2019). Legumes show similar patterns: lentil and faba bean under FACE had even lower grain Fe and Zn when eCO_2_ was combined with drought (Parvin et al., 2019). These findings, consistent with the broader multi-crop dataset compiled by Dietterich et al. (2015), suggest that rising CO_2_ may worsen hidden hunger, unless we also protect crop nutritional quality alongside yield.

Despite this evidence, we do not fully understand how eCO_2_ lowers micronutrient content, and many interacting mechanisms have been proposed (Myers et al., 2014). First, partial stomatal closure under eCO_2_ reduces transpiration and weakens mineral movement from the soil to the plant. Second, eCO_2_ may change how cell membranes take up, move, and redistribute micronutrients, including proteins that handle Fe, Zn, Mn, and Cu. Third, a dilution effect may occur: carbohydrates and grain yield increase faster than mineral uptake and loading, lowering micronutrient levels per unit dry mass. These processes interact, and factors such as rhizosphere chemistry, nitrogen levels, soil pH, mycorrhizal relationships, genotype, and stresses like drought and heat shape the results, explaining why micronutrient decline varies across crops, environments, and experiments (Ainsworth and Long, 2021). An integrated, mechanistic synthesis is still needed.

This review addresses that gap. It focuses on micronutrients most critical for human and crop health principally Fe, Zn, Cu, and Mn and, where evidence permits, also considers boron, molybdenum, selenium, and iodine. Specifically, the review (i) synthesises how plants acquire, transport, and load micronutrients under eCO_2_ and related climate stresses; (ii) compares crop- and element-specific responses reported in FACE studies, controlled-environment experiments, and meta-analyses; (iii) evaluates how co-occurring stresses heat, drought, low nitrogen, poor soil, and ozone modify these responses; and (iv) identifies key research gaps and evaluates breeding, agronomic biofortification, and soil-microbial strategies for safeguarding grain mineral quality under future CO_2_ conditions. While Ebi and Loladze (2019) focused broadly on how climate change and eCO_2_ jointly compromise crop yields and nutritional quality noting reductions of 5–15% in protein and minerals and up to 30% in B vitamins, the present review differs from prior work by moving beyond broad commentary to a mechanistic, element-resolved synthesis: it quantifies eCO_2_ effects across eight elements (Fe, Zn, Cu, Mn, B, Mo, Se, I) rather than protein and minerals in aggregate; incorporates FACE and meta-analytic evidence published since 2019; explicitly traces the membrane-transport and transpiration-driven mass-flow mechanisms underlying dilution; and integrates breeding, agronomic, and soil-microbial mitigation strategies within a single framework. **Table 1** positions this review against prior syntheses on this topic. By linking crop physiology to nutritional security, this review aims to provide researchers, breeders, and policymakers with a clear, actionable framework at the intersection of climate change, food quality, and public health.

**Table 1:** Positioning of the present review relative to prior syntheses on climate change, eCO_2_, and crop nutritional quality.

Prior review (author, year, journal)	Primary scope	Micronutrients/elements addressed	Literature coverage	Gap addressed by the present review
Myers et al. (2014), Nature	FACE-based meta-analysis quantifying eCO_2_ effects on grain nutrient content across major C3 crops	Zn, Fe, protein (aggregate)	Through 2013	Landmark quantitative baseline restricted to Zn, Fe and protein, with no treatment of Cu, Mn, B, Mo, Se, I or the underlying transport mechanisms; present review extends coverage to eight elements within a mechanistic framework
Soares et al. (2019), Plant and Soil	Narrative review of multiple climate stressors (eCO_2_, temperature, salinity, waterlogging, drought) on crop nutrient quality and mitigation	General minerals and macronutrients; not element-resolved	Through 2018	Breadth across five stressors limits depth on eCO_2_-specific physiology and precludes quantitative, element-by-element synthesis; present review narrows focus to eCO_2_ and reports element-level magnitude estimates
Ebi and Loladze (2019), Lancet Planetary Health	Commentary linking climate-driven yield loss and CO_2_-driven nutrient decline to global food-security and hidden-hunger burden	Protein, minerals, B vitamins (aggregate percentage ranges)	Through 2018	Short-format commentary without crop- or element-specific mechanistic detail or primary-literature synthesis; present review provides the full-length, evidence-based treatment
Scarpa et al. (2020), Environmental Research Letters	Systematic map of empirical literature linking general climatic drivers (not eCO_2_ specifically) to food nutrient content	Macronutrients and micronutrients broadly, across terrestrial and aquatic food sources	2013–2019	Maps the evidence landscape rather than synthesising findings, and is not focused on eCO_2_ or crop physiology; present review moves from mapping to mechanistic, quantitative synthesis
Ainsworth et al. (2025), Phil. Trans. R. Soc. B	Narrative review of physiological mechanisms linking rising [CO2] to crop yield gains and nutritional quality declines, with emphasis on biofortification potential	Protein and micronutrients broadly (not element-resolved)	Through 2024	Physiological/mechanistic framing without quantitative, element-by-element magnitude estimates or multifactor (drought/N/heat) interaction analysis; present review adds quantitative element-level synthesis and cross-stressor interaction focus
Ekele et al. (2025), Stress Biology	Wheat-focused review of eCO2 impacts on productivity, water/nutrient-use efficiency, and nutritional quality, with attention to heat and drought interactions	N, minerals, vitamins, polyphenols (wheat-specific)	Through 2024	Single-crop (wheat) focus limits generalisability across cereal and legume systems; present review synthesises evidence across multiple staple crops and functional groups
Wang et al. (2025), Frontiers in Plant Science	Meta-analysis of eCO2 effects on Zn and Fe in vegetable crops specifically	Zn, Fe (vegetables only)	Through 2024	Restricted to vegetable crops rather than staple cereals/legumes that dominate global caloric and micronutrient intake; present review focuses on grain-bearing staples most relevant to hidden-hunger burden
ter Haar et al. (2025), Global Change Biology	Largest meta-analysis to date of direct eCO2 stoichiometric effects on edible plant parts (5,324 entries, 43 crops, 32 nutrients/antinutrients), including both C3 and C4 species	Broad ionome (32 elements/antinutrients, incl. Zn, Fe, and others)	Through 2024/2025	Exceptionally broad quantitative coverage but not organised around transport mechanisms, multifactor field interactions, or mitigation strategy; present review adds mechanistic transport framework and integrated mitigation synthesis
Walsh and Lundgren, (2024), Plants, People, Planet	Compares nutritional-quality responses of C3 versus C4 crops under future climate scenarios	General nutrient/quality traits, contrasted by photosynthetic pathway	Through 2023	Organised around C3/C4 comparison rather than transport physiology or mitigation strategy; present review adds explicit membrane-transport detail and an integrated mitigation framework
Present review	eCO_2_-specific, mechanistic and quantitative synthesis of grain micronutrient dilution, integrating FACE evidence with mitigation strategy	Fe, Zn, Cu, Mn, B, Mo, Se, I	Through 2024	—

Hidden hunger — A form of undernutrition caused by chronically inadequate intake of essential vitamins and minerals (e.g., Fe, Zn, vitamin A), which can occur even when total caloric/energy intake is sufficient and often produces no overt clinical signs (FAO, 2013).

Micronutrient deficiency — The clinical/physiological state that results when the body’s intake or absorption of a specific vitamin or mineral falls below the level needed for normal function; hidden hunger is the population-level, often subclinical manifestation of widespread micronutrient deficiency (Bailey et al., 2015).

Carbon–nutrient dilution effect — The reduction in grain mineral *concentration* that occurs when elevated CO_2_ increases carbohydrate/biomass accumulation faster than mineral uptake and loading keep pace, so the same (or even a larger) amount of mineral is distributed across more dry matter (Loladze., 2014).

Bioavailability — The proportion of an ingested mineral (e.g., Fe, Zn) that the human body can actually absorb and utilize, as distinct from its total concentration in the food; it is influenced by antinutritional compounds such as phytate, the food matrix, and interactions with other dietary components (Gibson et al., 2010).

Biofortification — The process of increasing the density of bioavailable micronutrients in edible crop tissues, whether through conventional breeding, transgenic approaches, or agronomic practices (e.g., foliar or soil mineral application), to improve nutritional outcomes at the population level (Bouis and Saltzman, 2017).

## 2 Physiological drivers of micronutrient dilution under elevated CO_2_

Understanding why elevated CO_2_ (eCO_2_) lowers grain micronutrient levels means tracing several linked physiological processes. It starts with increased carbon fixation and includes changes in stomatal conductance, transpiration, root nutrient acquisition, rhizosphere nutrient movement, and internal mineral allocation (McGrath and Lobell, 2013). The problem is not necessarily a reduction in total nutrient absorption alone; rather, several mechanisms can contribute simultaneously, including carbohydrate dilution, reduced transpiration-driven nutrient mass flow, and altered nutrient allocation within the plant (McGrath and Lobell, 2013). More broadly, carbon gain can increase faster than mineral accumulation, resulting in a dilution of mineral concentrations as carbohydrate production increases under eCO_2_ (McGrath and Lobell, 2013). As a result, grains may accumulate more carbohydrates without a proportional increase in essential minerals, leading to lower concentrations of nutrients such as Fe and Zn in edible tissues (McGrath and Lobell, 2013; Myers et al., 2014). Experimental evidence from multiple crops confirms that elevated CO_2_ can reduce grain concentrations of Fe and Zn, raising concerns about the nutritional quality of staple crops (Myers et al., 2014). **Figure 1** provides a conceptual summary of the interacting physiological processes through which eCO_2_ promotes grain micronutrient dilution.

**Figure 1:**
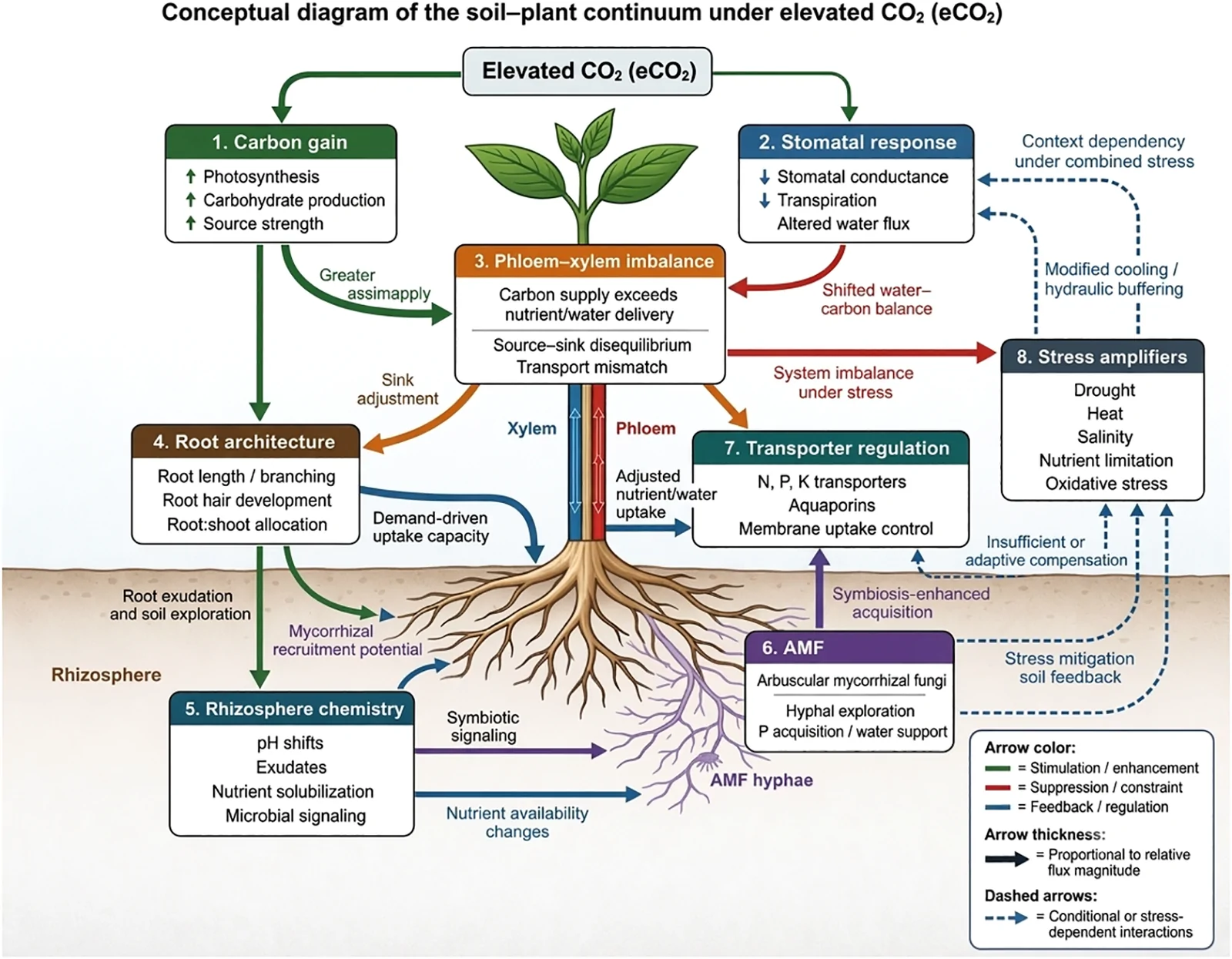
Conceptual overview of micronutrient dilution under eCO_2_ in staple crops. This image summarizes the major pathways through which eCO_2_ reduces grain micronutrient concentration. eCO_2_ enhances photosynthesis and carbohydrate accumulation, but this gain is not matched by proportional increases in mineral uptake, transport, and grain loading. Reduced stomatal conductance lowers transpiration-driven mass flow of nutrients to roots, while changes in transporter activity, rhizosphere processes, and internal partitioning further constrain the delivery of Fe, Zn, Cu, and Mn to developing grain. The image integrates these interacting processes to show how eCO_2_ shifts the carbon-to-mineral balance in favor of yield quantity over nutritional quality. The magnitude of micronutrient responses is context dependent and varies with crop species, genotype, CO_2_ concentration, nutrient availability, and environmental conditions; therefore, the image represents a conceptual framework rather than a quantitative estimate of micronutrient decline.

### 2.1 Carbon surplus and the dilution effect

In C3 crops, eCO2 generally enhances net photosynthesis by increasing substrate availability for *Rubisco* and suppressing photorespiration, especially under non-stress conditions (Ainsworth and Rogers, 2007). This outcome often stimulates biomass production and grain yield, but the increase in carbon assimilation is seldom matched by a proportional rise in mineral uptake and loading (McGrath and Lobell, 2013). As a result, grain mineral concentration declines because carbohydrate and dry matter accumulate faster than micronutrients (McGrath and Lobell, 2013). This carbon–nutrient dilution effect is now widely regarded as one of the principal explanations for lower grain Fe and Zn concentrations under eCO2 (Dietterich et al., 2015; Myers et al., 2014). Importantly, dilution does not always imply that total mineral uptake per plant decreases; in many cases, mineral accumulation simply fails to keep pace with the enlarged carbon pool (McGrath and Lobell, 2013). This distinction is critical from a management perspective because strategies intended to preserve nutritional quality must improve mineral uptake efficiency and grain loading, rather than merely increasing vegetative growth (Ainsworth and Rogers, 2007; Myers et al., 2014; McGrath and Lobell, 2013).

### 2.2 Closing of stomata and reduced transpiration-driven mass flow

Partial guard cell closure is one of the most consistent plant responses to eCO_2_. Reduced stomatal aperture lowers stomatal conductance and frequently decreases transpiration at both leaf and canopy scales. Because many mineral nutrients reach the root surface partly through transpiration-driven mass flow, reduced water movement can restrict nutrient movement from the bulk soil solution to uptake sites (Ainsworth and Rogers, 2007; Leakey et al., 2009). Evidence from wheat FACE and glasshouse experiments shows that eCO_2_ reduces both transpiration and the xylem-sap concentration of relatively mobile cations such as Ca and Mg, consistent with a transpiration-driven mass-flow constraint (Houshmandfar et al., 2015). However, a companion study using the same experimental system found that aboveground uptake of most micronutrients was not reduced, and in several cases increased, under eCO_2_ weakening the case that reduced transpiration alone explains declining tissue mineral concentration (Houshmandfar et al., 2018). Reduced mass flow therefore appears to constrain delivery of specific, xylem-mobile nutrients, but its role in the Fe and Zn decline emphasized elsewhere in this review is less well resolved than for Ca and Mg.

### 2.3 Root system architecture shifts

eCO_2_ often increases carbon allocation below ground and can stimulate root proliferation, although the response varies with species, genotype, soil profile, and environmental conditions. FACE-based evidence suggests that eCO_2_ may promote greater root length and altered root distribution, likely improving soil exploration. However, a larger root system does not necessarily guarantee improved micronutrient nutrition. Its effectiveness depends on the nutrient status of the soil zones explored, the metabolic cost of maintaining additional root tissue, and the extent to which enhanced root growth is accompanied by sufficient transporter activity and physiological demand for mineral uptake. Thus, root proliferation under eCO_2_ may improve access to soil resources in some situations, but it does not consistently compensate for reduced transpiration-driven nutrient supply or restrictions in internal mineral transport to grain (Bourgault et al., 2021).

### 2.4 Rhizosphere chemistry and exudation

eCO_2_ also alters rhizosphere function by increasing carbon flow below ground and changing the quantity and composition of root exudates. Greater release of soluble carbon compounds can influence microbial activity, nutrient solubility, and root–soil chemical interactions. In principle, these changes may either enhance or constrain micronutrient acquisition depending on soil pH, nutrient form, microbial composition, and crop species (Jo et al., 2022). For Fe and Zn in particular, rhizosphere responses to eCO_2_ are highly context-dependent, and broad generalisations are difficult to justify across contrasting soils. It is therefore more appropriate to treat rhizosphere modification as a variable, soil-dependent component of the eCO_2_ response rather than a uniformly beneficial or harmful mechanism (Dong et al., 2021).

### 2.5 Arbuscular mycorrhizal fungi and microbial feedback

The greater carbon supply to roots under eCO2 may also affect symbiotic and free-living soil microorganisms, including arbuscular mycorrhizal fungi (AMF) (Smith and Read, 2008). These fungi are especially relevant to micronutrient nutrition because they extend the effective absorptive zone beyond the root depletion zone and can improve the acquisition of relatively immobile nutrients, particularly Zn and, in some circumstances, Fe (Smith and Read, 2008; Coccina et al., 2019). Experimental evidence indicates that the mycorrhizal pathway can contribute appreciably to grain Zn accumulation in cereals (Coccina et al., 2019). However, the magnitude of this benefit depends strongly on host genotype, phosphorus status, soil fertility, and environmental stress (Smith and Read, 2008; Coccina et al., 2019). Under eCO2, AMF and associated microbial feedback may therefore buffer micronutrient dilution in some systems, but they do not consistently eliminate it (Coccina et al., 2019; Al-Hadeethi et al., 2019). Their role is best viewed as conditional and strongly dependent on the wider soil–plant environment (Smith and Read, 2008; Coccina et al., 2019; Al-Hadeethi et al., 2019).

### 2.6 Transporter regulation and internal mineral partitioning

In addition to whole-plant water relations and rhizosphere effects, micronutrient dilution under eCO_2_ has a molecular dimension. The uptake, translocation, remobilisation, and grain loading of Fe, Zn, Mn, and Cu depend on coordinated transporter systems, including members of the *IRT, ZIP, NRAMP, YSL*, and related families. There is increasing evidence that eCO_2_ can alter the expression of genes involved in nutrient uptake and partitioning, although the strength and direction of these responses differ across crops and mineral elements (Jo et al., 2022). At present, the strongest conclusion is not that eCO_2_ triggers a single universal transporter response, but that altered carbon status, altered nutrient demand, and interactions with nitrogen and water supply can reshape transporter regulation and internal metal allocation. This remains one of the least resolved mechanistic areas, particularly for Mn and Cu, and future work linking gene expression, transporter activity, and mineral fluxes during grain filling will be essential (Roy et al., 2022). As shown in **Figure 2**, micronutrient decline under eCO_2_ is linked not only to reduced external supply but also to constraints in internal transport and grain loading.

**Figure 2:**
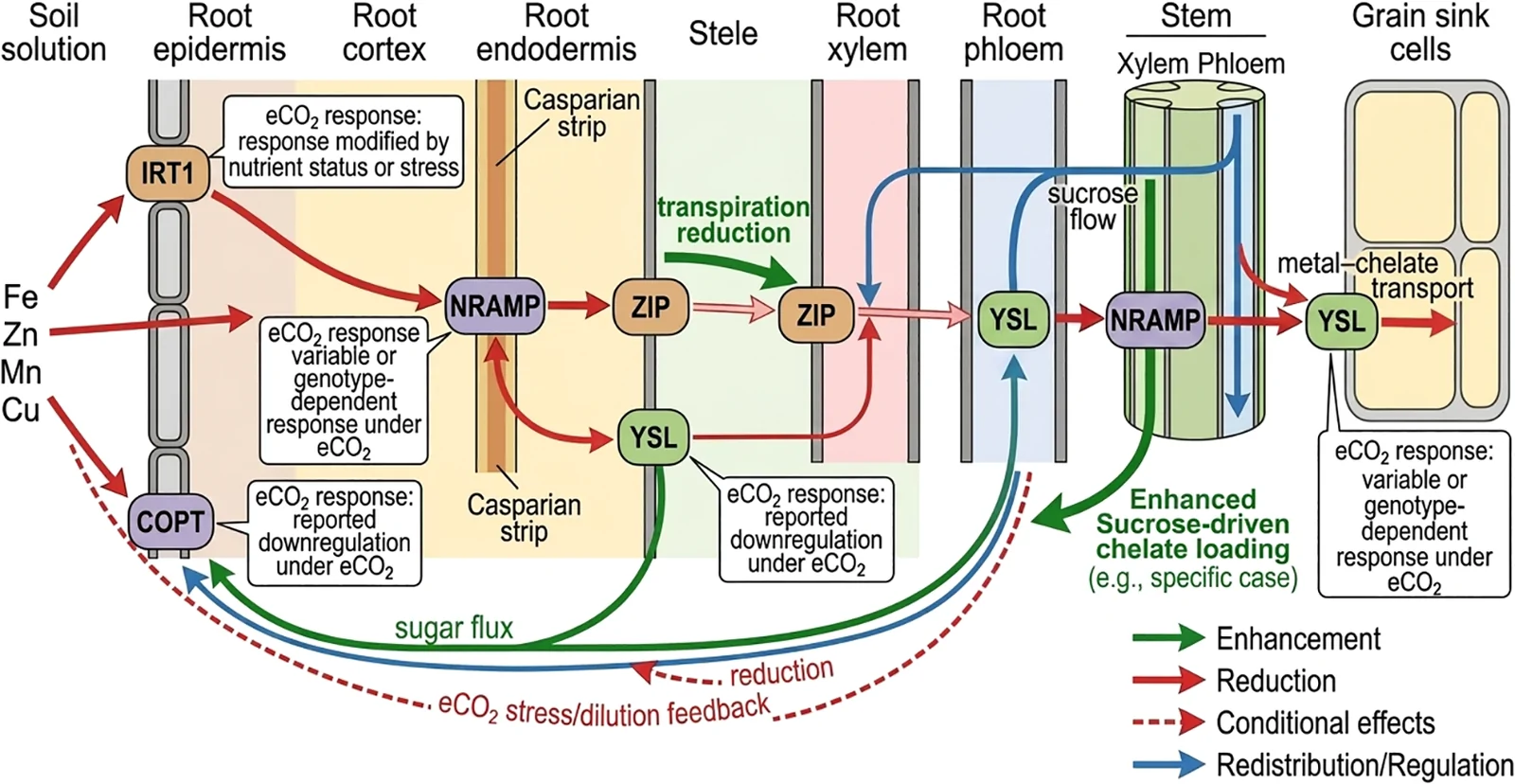
Root uptake, transport, and grain loading network for micronutrients under eCO_2_. This image illustrates the principal transport pathways involved in micronutrient acquisition and redistribution under eCO_2_, including uptake at the root surface, radial transport through root tissues, xylem and phloem translocation, and final loading into grain. Key transporter families associated with Fe, Zn, Mn, and Cu homeostasis, including *IRT, ZIP, YSL, NRAMP*, and *COPT*, are positioned within the root–shoot–grain continuum to highlight their functional roles. The image also indicates how eCO_2_ may alter these pathways through reduced mass flow, modified root physiology, and changes in internal remobilization during grain filling. **Representative transporter genes, including *OsIRT1* and members of the *OsZIP* family, are discussed in the accompanying text to provide molecular context. Transporter responses may vary among species, genotypes, tissues, and experimental conditions; therefore, the image emphasizes principal transport pathways rather than assigning a uniform up- or down-regulation pattern to all transporters.

Among the transporter families discussed above, IRT1 and members of the ZIP family are the most consistently implicated in the eCO_2_ response, given their central role in Fe and Zn uptake at the root epidermis and their frequent appearance in transcriptomic studies of CO_2_-exposed roots (Jo et al., 2022); NRAMP and YSL responses are reported less consistently and appear more species-dependent. Comparisons between rice and wheat suggest that the direction and magnitude of transporter regulation under eCO_2_ are not conserved across these two crops, consistent with the broader element-by-element variability described in Section 3. The developmental timing of CO_2_ exposure is also likely to matter: transporter changes during vegetative growth influence root uptake capacity, whereas changes during reproductive development influence remobilisation and grain loading directly, and current studies rarely distinguish between these two windows.

### 2.7 Compound effects under co-occurring stresses

Under field conditions, eCO_2_ rarely acts alone. Warming can accelerate crop development and shorten the duration of grain filling, thereby limiting the time available for mineral accumulation in grain (Wang and Liu, 2021). Drought intensifies the problem by further reducing xylem water flow and mass transport from the soil to the root (Parvin et al., 2019; Wang and Liu, 2021). Nitrogen limitation can aggravate the imbalance between carbon gain and nutrient acquisition, while salinity and other soil constraints may interfere with uptake processes and ionic homeostasis (Wang and Liu, 2021). Evidence from both legumes and cereals indicates that micronutrient losses are often greater when eCO_2_ is combined with drought or heat than when CO_2_ enrichment occurs in isolation (Parvin et al., 2019). This interaction is especially important for future food-quality projections because it suggests that the nutritional penalty of rising atmospheric CO_2_ may become more severe in the same environments already exposed to increasing climatic stress (Parvin et al., 2019; Wang and Liu, 2021).

## 3 Element-by-element synthesis of grain micronutrient responses under elevated CO_2_

The physiological mechanisms outlined in Section II translate into element-specific outcomes because each micronutrient differs in soil mobility, root uptake pathway, vascular transport behaviour, and remobilisation during grain filling. As a result, eCO_2_ does not affect all elements equally. The clearest and most consistent declines are observed for Fe and Zn, whereas responses of Cu, Mn, B, Mo, Se, and I are more variable and, in several cases, still constrained by limited field evidence. **Table 2** summarises the best-supported cross-crop patterns (Dietterich et al., 2015). Taken together, the available data indicate that rising atmospheric CO_2_ poses its greatest nutritional risk through declines in grain Fe and Zn, with other micronutrients showing either weaker, more context-dependent, or still insufficiently resolved responses.

**Table 2:** Summary of grain micronutrient concentration responses to elevated CO_2_ (≥550 ppm) across major crop species.

Element	Crop(s)	Experimental system	Mean change in grain concentration	Principal mechanism(s)	Key reference(s)	Number of studies
Fe	Wheat	FACE/OTC	↓, commonly about 5–15%	Carbon–nutrient dilution; reduced transpiration-driven mass flow; altered nutrient allocation	Myers et al. (2014); McGrath and Lobell (2013); Högy et al. (2009)	3
Fe	Rice	FACE + chamber	↓, commonly >10%	Dilution; reduced transpirational supply; impaired grain loading	Chumley and Hewlings (2020)	1
Fe	Lentil, faba bean	FACE	−4 to −25%	Mass-flow limitation plus yield dilution; drought amplifies decline	Parvin et al. (2019)	1
Zn	Wheat	FACE	↓, typically about 9–12%	Carbon–nutrient dilution; reduced transpiration; altered allocation	Ainsworth and Long (2021); Myers et al. (2014)	2
Zn	Wheat, rice, maize, field pea, soybean	FACE + chamber + meta-analysis	↓, about 3–12% overall; soybean often non-significant	Dilution plus reduced nutrient delivery and altered uptake/partitioning	Al-Hadeethi et al. (2019)	1
Zn	Lentil, faba bean	FACE	−4 to −25%	Mass-flow limitation plus yield dilution; drought amplifies decline	Parvin et al. (2019)	1
Cu	Cereals and legumes	Limited evidence	Variable; no robust cross-crop consensus	Likely affected by dilution and altered uptake/partitioning, but evidence remains sparse	Wang and Liu (2021)	1
Mn	Wheat and some cereals	FACE + chamber	Mixed; often unchanged to slightly decreased, occasionally increased	Soil chemistry and allocation effects; response is context dependent	Högy et al. (2009); McGrath and Lobell (2013)	2
B	Multiple species	Mostly chamber	Variable; insufficient evidence for a robust general estimate	Species- and B-status-dependent; possible dilution in some systems	Soares et al. (2021); Kaur et al. (2025)	2
Mo	Cereals and legumes	Very limited evidence	Variable; insufficient evidence for a robust general estimate	May reflect N metabolism and allocation, but evidence is too sparse for firm synthesis	Hungate et al. (2004)	1
Se	Rice and some non-cereal systems	Sparse	Insufficient evidence for a robust cross-crop estimate	Possible dilution/allocation effects, but dataset is limited	Narrative discussion only	0
I	Multiple species	—	Data gap	Plausible dependence on xylem transport, but direct eCO_2_ evidence is lacking	Data gap	0

### 3.1 Iron

Iron concentration in grain generally declines under eCO_2_ across cereals and, under some conditions, legumes, although the magnitude of decline varies with genotype, soil properties, water supply, and experimental system. In wheat, FACE and chamber studies commonly report reductions in grain Fe concentration, with declines often in the range of 5–15% and occasionally exceeding this under particular environments or management conditions (Högy et al., 2009; Myers et al., 2014). In rice, the available evidence also points to a consistent decline. A systematic review of high-quality studies concluded that eCO_2_ commonly reduces grain Fe concentration, with losses frequently exceeding 10% near the CO_2_ concentrations expected by mid-century (Chumley and Hewlings, 2020; Hu et al., 2022). Grain legumes show a similar overall pattern but with stronger dependence on co-occurring drought. In lentil and faba bean grown under FACE conditions, grain Fe declined modestly under well-watered conditions but fell much more sharply when eCO_2_ was combined with water limitation, pointing to the strong interaction between carbon enrichment and reduced mass flow to the root surface (Parvin et al., 2019). Collectively, these results show that Fe decline is one of the most robust nutritional consequences of eCO_2_ and is driven by the combined effects of dilution, reduced transpiration-linked nutrient delivery, and constrained grain loading (McGrath and Lobell, 2013; Wang and Liu, 2021). Fe decline is one of the most robust and mechanistically well-supported micronutrient responses to eCO_2_ identified in this review.

### 3.2 Zinc

Among all micronutrients, Zn shows the most consistent and quantitatively important decline under eCO_2_. A meta-analysis spanning multiple crops and experimental platforms found grain Zn reductions of roughly 3–12% at CO_2_ concentrations of 550 ppm or higher, with soybean representing a partial exception because responses there were often small or statistically non-significant (Al-Hadeethi et al., 2019). In wheat, FACE-based evidence indicates a recurrent decline of around 9–12%, a magnitude large enough to be nutritionally important in populations that depend heavily on cereal staples for daily Zn intake (Ainsworth and Long, 2021; Myers et al., 2014). Legume responses again become more severe under drought, with lentil and faba bean showing substantially greater Zn losses when eCO_2_ is accompanied by seasonal water deficit (Parvin et al., 2019). The consistency of the Zn response across cereals, the support from both field and meta-analytic evidence, and its high relevance to human nutrition make Zn the clearest example of climate-driven grain micronutrient dilution under eCO_2_. Together with Fe, Zn therefore anchors the clearest and best-supported case for CO_2_-driven micronutrient decline in this review, a conclusion echoed throughout the sections that follow.

### 3.3 Copper

Copper responses are less consistent than those of Fe and Zn, and the available evidence is still too limited to support a strong cross-crop quantitative generalisation (Wang and Liu, 2021). Some studies report modest declines in grain Cu concentration under eCO_2_, whereas others show little change, suggesting that species, soil conditions, and co-occurring stresses strongly influence the outcome (Wang and Liu, 2021). At present, the most defensible conclusion is that Cu may decline in some cereal systems through the same broad processes that affect Fe and Zn, namely dilution and altered uptake or partitioning, but that the evidence base remains sparse and too uneven for a firm general estimate (Wang and Liu, 2021). This is especially true under realistic field conditions, where multi-site FACE studies reporting grain Cu are still uncommon (Wang and Liu, 2021). Accordingly, Cu should be treated as a potentially responsive but insufficiently resolved micronutrient in the current eCO_2_ literature (Wang and Liu, 2021). This weaker and less consistent signal stands in clear contrast to the robust declines documented for Fe and Zn above.

### 3.4 Manganese

Manganese differs from Fe and Zn in showing a less uniform response to eCO2 (Wang et al., 2020). Available studies suggest that grain Mn is often unchanged, sometimes slightly decreased, and in some systems even increased, showing a stronger dependence on soil chemistry and element-specific transport dynamics than is seen for Fe or Zn (Högy et al., 2009; Wang et al., 2020). In wheat, FACE evidence has shown that eCO2 can reduce some mineral concentrations while leaving others less affected, and more recent field work in rice–wheat systems indicates that Mn may even increase under certain combinations of eCO2 and warming (Högy et al., 2009; Wang et al., 2020). These mixed outcomes suggest that Mn is partly buffered by its distinct geochemical behaviour in soil and by context-dependent changes in rhizosphere chemistry (Wang et al., 2020). Even so, the existing dataset is not yet broad enough to define a single universal direction of response across crops and environments (Wang et al., 2020). It is therefore safest to interpret Mn as a comparatively less sensitive, but still variable, component of the grain ionome under eCO2 (Högy et al., 2009; Wang et al., 2020). As with Cu, this variability sets Mn apart from the strong and consistent Fe and Zn responses, reinforcing that CO2-driven micronutrient risk is not uniform across elements (Högy et al., 2009; Wang et al., 2020).

### 3.5 Boron and molybdenum

For B, the evidence base is smaller than for Fe, Zn, Cu, or Mn, but it is no longer empty. The clearest direct evidence comes from FACE studies on soybean, where grain B concentration declined by an average of 18.5% under eCO_2_ across thirteen genotypes, with individual genotypes losing between 20.9% and 42.8% (Soares et al., 2021). A separate open-top-chamber study similarly reported reduced seed B alongside Fe, Zn, and Mn under eCO_2_ in soybean (Kaur et al., 2025). These declines are broadly consistent with a carbon-dilution mechanism, in which B accumulation fails to keep pace with grain biomass, but responses vary strongly with genotype and soil B availability, and equivalent FACE data for cereals are still lacking.

For Mo, direct grain-level evidence under eCO_2_ remains scarce, but the clearest mechanistic signal comes from ecosystem-scale FACE work rather than grain-focused studies. In a long-term Florida scrub-oak FACE experiment, eCO_2_ initially stimulated legume nitrogen fixation, but this stimulation declined over successive years and reversed as molybdenum availability became limiting, implicating reduced Mo bioavailability as CO_2_ rose (Hungate et al., 2004). Because Mo is a cofactor for nitrogenase and nitrate reductase, this pattern is consistent with the broader hypothesis that eCO_2_ constrains Mo-dependent nitrogen metabolism, particularly in legumes, more than it drives simple carbon dilution of grain Mo. Grain-level FACE datasets that directly quantify Mo concentration in staple cereals and legumes remain scarce and are identified here as a priority for future FACE and open-top-chamber studies (Section 6). Taken together, B and Mo should no longer be treated as entirely unresolved: available evidence points toward a decline in B under eCO_2_, at least in soybean, and toward Mo-limitation effects on nitrogen metabolism, but robust cross-crop, cross-experiment quantification for both elements is still lacking. Despite this thinner evidence base, B and Mo should not be dismissed from future climate-nutrition research agendas, particularly given their functional importance in seed development and nitrogen metabolism.

### 3.6 Selenium and iodine

Selenium and iodine are particularly important for human nutrition, but evidence for their effects on grain yield under eCO_2_ remains sparse. Existing studies suggest that Se concentration may decline in some crop systems, yet the dataset is too limited and fragmented across species and environments to support a broad quantitative conclusion. The evidence gap is even larger for I. Because iodine movement in plants is closely associated with water flow in the xylem, a reduction in transpiration under eCO_2_ could plausibly reduce shoot and grain iodine delivery, but direct field evidence remains largely absent. These two elements, therefore, represent an important frontier for future work, particularly because hidden-hunger assessments that focus solely on Fe and Zn may underestimate the wider nutritional consequences of rising atmospheric CO_2_. The nutritional stakes of these gaps are high. Selenium is a component of selenoproteins, including glutathione peroxidases, and is central to antioxidant defense and immune function, so even modest dietary shortfalls can have measurable health consequences in populations already close to deficiency thresholds (Rayman., 2012). Iodine is required for thyroid hormone synthesis, and inadequate intake is a leading preventable cause of impaired cognitive development; populations in inland regions far from marine dietary iodine sources are disproportionately dependent on soil- and crop-derived iodine, and are therefore potentially more exposed if transpiration-linked iodine delivery to grain declines under eCO_2_ (Gonzali et al., 2017).

Considered alongside the other elements reviewed above, this element-by-element evidence points to a clear hierarchy of CO_2_ sensitivity: Fe and Zn decline most consistently and are best supported by FACE, systematic review, and meta-analytic evidence; Cu and Mn are more variable and context-dependent; and B, Mo, Se, and I remain too sparsely studied for firm quantitative conclusions, even though the underlying mechanisms carbon-nutrient dilution, reduced transpiration-driven transport, and altered uptake and grain loading plausibly extend to all seven elements. Overall, the literature therefore supports a central conclusion: eCO_2_ threatens grain nutritional quality most clearly through reductions in Fe and Zn, but the full micronutrient dimension of that threat is broader than current datasets allow us to quantify with confidence.

### 3.7 Phytate and bioavailability

Assessment of grain nutritional quality based solely on total mineral concentration may overestimate the nutritional benefits of Fe and Zn, because their bioavailability is strongly influenced by antinutritional compounds, particularly phytic acid (phytate). Phytate is a major phosphorus-storage compound in cereals and legumes and can form relatively insoluble complexes with Fe, Zn and Ca, reducing their solubility and intestinal absorption (Frossard et al., 2000). Therefore, changes in grain Fe and Zn under eCO_2_ should be interpreted alongside phytate concentration and phytate molar ratios.

Evidence indicates that eCO_2_ can modify grain phytate concentration, although responses are crop-dependent. Guo et al. (2022) reported contrasting responses in rice and wheat under T-FACE conditions. In rice, eCO_2_ increased grain phytic acid (PA) by approximately 8%, together with increases in PA/Zn and other PA ratios. Under combined eCO_2_ and elevated temperature, phytic acid increased by 18.1%. In contrast, wheat showed reduced phytic acid under eCO_2_, with a 3.5% reduction under the combined treatment. These contrasting responses demonstrate that changes in mineral concentration alone cannot fully predict nutritional outcomes under eCO_2_.

Earlier work also reported reduced wheat grain phytate under eCO_2_. However, phytate ratios should be considered indicators rather than direct measures of human mineral absorption, because bioavailability is additionally influenced by the grain matrix, proteins and competing dietary components. This is particularly relevant in legumes, where phytate is an important determinant of Fe and Zn bioavailability (Zhang et al., 2022). Processing methods such as germination and fermentation can reduce phytate and improve mineral bioavailability, providing complementary post-harvest strategies.

## 4 Cross-factor interactions: how co-occurring stresses shape micronutrient dilution under elevated CO_2_

The element-level responses described in Section III do not occur in isolation under field conditions (Galani et al., 2022). In agricultural systems, eCO2 interacts with warming, drought, nutrient limitation, soil constraints, and atmospheric pollutants, producing outcomes that are frequently more complex than those observed in single-factor experiments (Galani et al., 2022). These interactions matter because grain micronutrient concentration is determined not only by carbon gain, but also by the timing of grain filling, water flux from soil to root, nutrient availability in the rhizosphere, and the plant’s ability to acquire, remobilise, and deposit minerals in reproductive sinks (Ekele et al., 2025). As a result, the nutritional consequences of eCO2 are best understood as the product of multiple interacting stresses rather than a simple CO2 effect alone (Galani et al., 2022).

### 4.1 eCO_2_ × elevated temperature

Temperature is one of the most important modifiers of crop response to eCO2 (Ekele et al., 2025). Warming can accelerate phenological development and shorten the duration of grain filling, thereby altering the time available for mineral accumulation in the grain (Chaturvedi et al., 2017). In wheat, combined eCO2 and heat are known to affect grain composition and end-use quality, including traits related to protein structure and baking performance (Ekele et al., 2025). However, the effect of warming on grain mineral concentration is not a simple amplification of the eCO2 penalty in all cases (Wang et al., 2025). Experimental evidence from wheat–rice systems show that eCO2 generally decreases grain nutrient concentrations, whereas canopy warming can, in some circumstances, increase grain mineral concentrations, partly by concentrating minerals in smaller grains or by modifying source–sink relations (Chaturvedi et al., 2017). This means that combined eCO2 and warming can yield mixed nutritional outcomes depending on species, cultivar, and environment, and should not be described as uniformly additive in the absence of crop-specific evidence (Chaturvedi et al., 2017).

### 4.2 eCO_2_ × drought and vapour pressure deficit

Drought remains one of the clearest co-stresses increasing the nutritional effects of eCO_2_ (Parvin et al., 2019). eCO_2_ alone commonly lowers stomatal conductance and transpiration, and under soil water deficit, this reduction in water flux can become even more pronounced (Parvin et al., 2020). Because transpiration contributes to the mass flow of several nutrients toward roots, reductions in water flux under both eCO_2_ and drought can constrain nutrient delivery and uptake (He and Dijkstra, 2014). This interaction is well illustrated in grain legumes (Parvin et al., 2019). In FACE-grown lentil and faba bean, grain Fe and Zn declined only modestly under well-watered conditions but dropped much more strongly under concurrent dry conditions, showing that water limitation can substantially magnify micronutrient dilution (Parvin et al., 2019). These findings suggest that future nutritional risks may be particularly pronounced where rising CO_2_ coincides with more frequent or severe drought during reproductive development (Parvin et al., 2019, 2020). Among the co-occurring stresses considered here, drought provides particularly clear evidence of an amplified eCO_2_-associated decline in grain micronutrient concentrations, especially when water limitation occurs during reproductive development (Parvin et al., 2019, 2020).

### 4.3 eCO_2_ × nitrogen and macronutrient supply

Nitrogen availability strongly modifies the grain-quality response to eCO_2_. When nitrogen supply is low, the imbalance between carbon gain and nitrogen acquisition can become more pronounced, potentially intensifying declines in grain protein and mineral nutrient concentrations (Igarashi et al., 2021; Ekele et al., 2025). A meta-analysis examining wheat, rice, maize, soybean, and field pea showed that the effects of elevated CO_2_ on grain protein, zinc, and iron were modified by nitrogen and water availability, with nutrient reductions generally being greater under less favourable nitrogen conditions (Al-Hadeethi et al., 2019). These results are consistent with FACE studies showing that elevated CO_2_ can reduce grain nitrogen and zinc concentrations across crops and soils (Myers et al., 2014). This interaction is agronomically important because nitrogen limitation can constrain plant nitrogen uptake, assimilation, growth, and the subsequent transfer of nutrients to developing grains (Igarashi et al., 2021; Chakwizira et al., 2024). Thus, nitrogen management may be an important practical strategy for reducing, although not necessarily eliminating, the nutritional penalty of eCO_2_ in cereal-based systems. Together with drought, nitrogen limitation should therefore be considered a major modifier of eCO_2_ effects on grain nutritional quality (Al-Hadeethi et al., 2019).

### 4.4 eCO_2_ × soil constraints and long-term soil context

Soil conditions can either moderate or intensify micronutrient responses to eCO_2_, but the direction of the effect depends on the underlying fertility context. Long-term FACE evidence indicates that eCO_2_ lowers grain nutrient concentrations more strongly in relatively fertile soils than in infertile soils, suggesting that soil background conditions shape the magnitude of nutritional decline (Jin et al., 2019). This finding is important because it shows that the grain nutrient response cannot be predicted solely from atmospheric change. Instead, soil fertility status, nutrient balance, and long-term plant–soil feedbacks must be considered together. More broadly, calcareous soils, acidic soils, and saline environments may each modify the availability and mobility of Fe, Zn, Mn, and other minerals in different ways, but the current evidence base is still too limited to support a universal quantitative rule for these interactions (Jin et al., 2019). At present, the most defensible conclusion is that soil context matters greatly, but its effects remain strongly site specific.

### 4.5 eCO_2_ × tropospheric ozone

Tropospheric ozone adds one more layer of complexity. eCO_2_ can partially offset ozone-induced yield loss by supporting photosynthesis, but this does not necessarily protect grain quality. In wheat, combined exposure to eCO_2_ and ozone has been shown to alter grain quality traits in ways that differ from those under either stress alone, indicating that the nutritional and technological quality responses cannot be inferred from yield response alone (Yadav et al., 2019). Ozone imposes oxidative stress and may therefore influence plant demand for micronutrients involved in redox metabolism, including Cu, Mn, and Zn (Sharma and Davis, 1997). Even so, the current evidence is insufficient to define a strong quantitative cross-crop effect of the eCO_2_ × ozone interaction on grain micronutrient concentration.

### 4.6 Functional-group contrasts: C_3_ vs C_4_, legumes, and crop diversity

Functional-group differences are highly relevant to nutritional resilience facing eCO_2_. C_4_ crops generally show a smaller direct photosynthetic response to eCO_2_ than C_3_ crops because they already concentrate CO_2_ around *Rubisco*. As a result, the classical carbon–nutrient dilution effect is often less pronounced in C_4_ grains than in C_3_ staples, although C_4_ crops may still benefit indirectly under drought through improved water relations. This distinction has important food-system implications because it suggests that crop diversification can moderate nutritional risk (Jobe et al., 2020). Legumes also occupy a special position. Under an adequate water supply, symbiotic nitrogen fixation can partly buffer the decline in grain nutrient concentration, but this buffering weakens under drought, where large declines in Fe and Zn can still occur. Together, these patterns support the idea that diversification toward nutritionally important legumes and resilient C_4_ crops may help reduce some of the grain-quality losses associated with rising atmospheric CO_2_. This resilience is, however, conditional rather than absolute: it can weaken substantially under concurrent drought or other co-occurring stress, as the legume evidence above illustrates. Emerging evidence reinforces this functional-group contrast but also cautions against treating it as absolute: while C_4_ species such as maize generally show little direct carbon-dilution response, several studies indicate that C_4_ grain mineral concentration can still be affected indirectly through altered water relations, canopy microclimate, and soil nutrient cycling under eCO_2_. The C_3_/C_4_ distinction is therefore best understood as a difference in degree and dominant mechanism direct carbon dilution in C_3_ crops versus indirect climate-mediated effects in C_4_ crops rather than a guarantee of C_4_ nutritional immunity (Jobe et al., 2020).

### 4.7 Multi-factor experiments and global nutritional projections

The need for multi-factor experimentation is now clear. Short-term or single-factor studies are useful for identifying mechanisms, but they do not completely capture the cumulative effects of eCO_2_ under realistic field conditions. Long-term FACE experiments have already shown that sustained CO_2_ enrichment can alter grain nutrient concentrations, depending on crop type and soil fertility. At the global scale, modelling studies indicate that the nutritional consequences are considerable. One widely cited assessment projected that eCO_2_ could place an additional 175 million people at risk of zinc deficiency by 2050, with the burden concentrated in areas already vulnerable to micronutrient malnutrition (Myers et al., 2015). Because most such projections are based mainly on direct eCO_2_ effects and cannot fully account for interacting co-stresses, they may still underestimate the field-relevant nutritional penalty in future climates (Loladze, 2014). Taken together, the cross-factor evidence reviewed in this section supports three conclusions. First, single-factor eCO_2_ experiments are mechanistically useful but often insufficient for field interpretation, since drought, nitrogen limitation, warming, and other constraints can substantially alter grain micronutrient outcomes (Abdelhakim et al., 2022). Second, the strongest and most consistent amplification occurs when eCO_2_ coincides with water or nitrogen limitation (Leakey et al., 2011). Third, crop functional diversity, particularly C4 species and well-performing legumes, offers partial but conditional resilience (Myers et al., 2014). Overall, the nutritional risk of rising atmospheric CO_2_ is likely to be shaped as much by its interactions with co-occurring stresses as by the direct CO_2_ effect itself, a picture that **Figure 3** summaries below.

**Figure 3:**
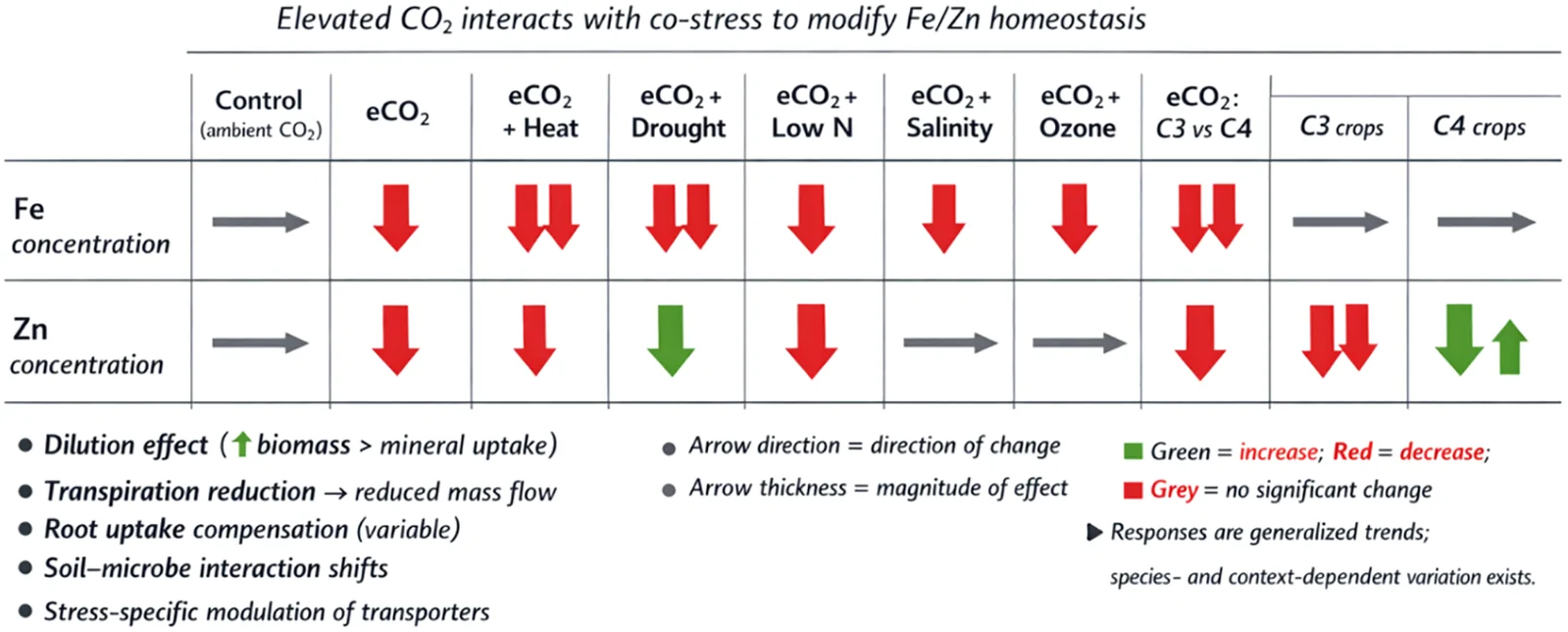
Element-specific grain micronutrient responses to eCO_2_ and interacting co-stresses. This image synthesizes the direction and relative magnitude of grain micronutrient responses under eCO_2_ alone and in combination with major co-stresses such as drought, warming, and low nitrogen supply. Fe and Zn show the most consistent declines across crops, whereas Cu and Mn responses are more variable and context dependent. The image also illustrates that co-stresses can modify, amplify, or attenuate the responses observed under eCO_2_ alone, highlighting the context dependence of micronutrient responses. Together, these patterns show that the severity of micronutrient dilution depends not only on the element itself but also on the environmental context in which crops are grown.

## 5 Methods and knowledge gaps: tools for decoding eCO_2_-driven micronutrient dilution

Progress in understanding how eCO_2_ alters grain micronutrient concentration has depended not only on advances in plant physiology but also on the strengths and limitations of the methods used to study the problem (Ainsworth and Long, 2005). Some approaches are powerful for capturing field-relevant outcomes, whereas others are better suited for elucidating mechanisms at the root, transporter, tissue, or model levels (Myers et al., 2014). A major challenge in this field is that no single method can simultaneously provide realistic crop responses, deep mechanistic resolution, and broad scalability (Long et al., 2006). For that reason, future progress will depend on combining complementary tools rather than relying on a single experimental platform. **Table 3** summarises the principal methodological trade-offs across the main approaches currently used in this research area.

**Table 3:** Methodological trade-offs for studying eCO_2_-driven micronutrient dilution.

Method	Field realism	Mechanistic depth	Cost	Scalability	Key gap addressed
FACE**/**FACE 2.0	High	Moderate	High	Low	Field-scale magnitude; multifactor interactions
Controlled chambers**/**OTCs	Low to moderate	High	Medium	Medium	Mechanism, dose response, controlled interaction testing
Stable isotope tracing	Medium	High	High	Low	Uptake versus remobilization; nutrient flux pathways
Root and rhizosphere imaging	Medium	High	High	Low	Spatial nutrient mobilization; root system dynamics
Multi-omics	Low to moderate	High	Medium	Medium	Transporter regulation; exudation and molecular responses
Crop–climate–nutrition modelling	Medium (projected)	Moderate	Low	High	Global nutritional risk; scenario comparison
Meta-analysis and synthesis	Medium	Low to moderate	Low	High	Cross-study quantification; moderator identification

### 5.1 Free-air CO_2_ enrichment and FACE 2.0

FACE remains the most robust field platform for assessing crop nutritional responses to eCO_2_ because it enriches CO_2_ levels under open-air conditions without imposing the artificial microclimatic constraints of enclosed systems (Ainsworth and Long, 2021). Much of the current evidence for declines in grain Fe and Zn under eCO_2_ has emerged from FACE experiments, particularly in major cereal and legume crops (Myers et al., 2014). The major strength of FACE resides in its field realism: crop canopies develop under natural light, wind, soil structure, and plant–soil–microbe interactions (Ainsworth and Long, 2005). This makes FACE indispensable for measuring the magnitude of micronutrient dilution in the real world. However, FACE experiments are expensive, technically demanding, and often limited in the number of sites, years, and factorial combinations that can be tested (Long et al., 2006). Newer multifactor systems, sometimes described as FACE 2.0, intend to address this drawback by combining eCO_2_ with warming, drought, and nutrient treatments (Mikkelsen et al., 2008). Such platforms are especially important because the nutritional effects of eCO_2_ are strongly modified by co-occurring stresses. The next major advance will require multi-year, multifactor FACE studies that combine grain ionomics with root traits, rhizosphere processes, and transporter-level measurements (Ainsworth and Long, 2021).

### 5.2 Controlled-environment chambers and open-top chambers

Controlled-environment and open-top chambers remain essential for mechanistic studies because they enable precise control over CO_2_ concentration, temperature, water supply, and nutrient availability (Ainsworth and Long, 2005). These systems are highly valuable when the aim is to isolate single variables, generate clear dose–response relationships, or test hypotheses related to transporter regulation, nutrient remobilisation, or elemental partitioning (Xue et al., 2015). They have also contributed substantially to the available information for less-studied elements that are not routinely measured in FACE programs. Their limitation, however, is that biological realism is reduced (McConnaughay et al., 1993). Chamber conditions can alter humidity, boundary-layer properties, transpiration, and rooting volume, whereas pot-based systems may not capture realistic rhizosphere chemistry or soil heterogeneity. As a result, chamber studies are best used to identify mechanisms and generate hypotheses, whereas field platforms are needed to determine whether those mechanisms remain important under agronomic conditions. In this field, chamber data should therefore be interpreted as mechanistic rather than definitive for predicting nutritional outcomes at the farm scale (Long et al., 2006).

### 5.3 Stable isotope and tracer techniques

Stable isotope and tracer methods provide one of the clearest ways to separate nutrient uptake, translocation, remobilization, and grain loading (Xue et al., 2015). Isotopes such as ^57^Fe and ^68^Zn can be used to track the movement of newly acquired mineral through plant tissues and into developing grain, allowing researchers to distinguish whether declines in grain concentration arise primarily from reduced uptake, altered internal redistribution, or weaker phloem loading during grain filling (Xue et al., 2015). When combined with elemental analysis, these approaches can move the field beyond descriptive concentration variations and toward true flux-based understanding (Xue et al., 2015). Water tracers can further strengthen interpretation by linking nutrient movement to transpiration-driven mass flow. In addition, spatially detailed elemental imaging of grain tissues can reveal whether eCO_2_ alters not only total mineral concentration but also the localisation of micronutrients within tissues relevant to nutritional bioavailability. At present, integrated tracer studies under joint eCO_2_, drought, and heat remain limited, and this is one of the clearest experimental gaps in the literature.

### 5.4 High-resolution root and rhizosphere phenotyping

Because many proposed mechanisms of micronutrient dilution are rooted in belowground processes, improved root and rhizosphere phenotyping is critical (Gray et al., 2013). Techniques such as minirhizotron imaging, X-ray computed tomography, and related non-destructive methods can reveal how eCO2 affects root proliferation, rooting depth, and the time dynamics of root activity (Mairhofer et al., 2013). At the rhizosphere scale, microelectrodes, optodes, and related sensors can help resolve changes in pH, oxygen status, and localised chemical conditions near the root surface (Blossfeld et al., 2013). These tools are especially relevant for testing hypotheses related to rhizosphere acidification, nutrient solubility, and the changing availability of Fe, Zn, and Mn under different soil conditions (Hinsinger et al., 2009). The major limitation is that these methods remain difficult to integrate into large-scale field experiments, notably under multifactor FACE conditions. Even so, combining root imaging with tracer approaches and soil chemical measurements would provide one of the most informative routes for resolving the belowground basis of micronutrient dilution.

### 5.5 Multi-omics and transporter network analysis

Transcriptomics, metabolomics, ionomics, and related multi-omics approaches provide an effective framework for understanding how eCO_2_ reshapes mineral uptake and partitioning at the molecular level (Baxter, 2010). In particular, transcriptomic analysis of roots and vascular tissues can clarify whether eCO_2_ alters the expression of transporters involved in Fe, Zn, Mn, and Cu homeostasis, and whether these responses depend on nitrogen status, water availability, or crop developmental stage (Liu et al., 2025). Metabolomic profiling of root exudates may also help reveal how carbon enrichment influences rhizosphere chemistry and micronutrient mobilisation (Bhavya et al., 2023). Yet these approaches have an important limitation: they often reveal correlation more readily than causation. Changes in transcript abundance do not automatically translate into changes in transporter activity or nutrient flux. For that reason, future studies should aim to connect multi-omics datasets with physiological measurements, isotope-based flux analysis, and functional validation in whole plants. The major frontier now is not the generation of more omics data alone, but the integration of molecular and physiological evidence into a coherent mechanistic framework.

### 5.6 Crop–climate–nutrition modelling

Process-based crop models and nutrition-linked assessment models remain essential for scaling plot-level findings to address regional and global food security questions. They provide the means to translate experimentally measured changes in grain nutrient concentration into projected shifts in dietary adequacy, population exposure, and future nutritional risk. This modelling step is particularly important because even modest percentage declines in grain Fe or Zn can have large public health consequences for populations that depend heavily on staple cereals (Myers et al., 2015). However, current models still carry substantial uncertainty. Root–soil mineral interactions, transporter kinetics, rhizosphere feedbacks, and the responses of less-studied elements such as Se, I, and Cu remain poorly represented. As a consequence, current global estimates of nutritional risk are useful but still incomplete. Advancing model performance will require stronger coupling between experimental records, soil information, crop physiology, and dietary evaluation systems (Smith and Myers, 2018; Beach et al., 2019).

More specifically, current crop–climate–nutrition models under-represent root–soil mineral interactions, transporter-level kinetics, and rhizosphere feedbacks, and they carry the least representation for the elements Se, I, Cu, B, and Mo for which the underlying experimental evidence base is itself the sparsest. Closing this gap will likely require a coordinated, multi-institutional modelling effort rather than incremental improvement of individual models in isolation. A structure similar to the Agricultural Model Intercomparison and Improvement Project (AgMIP), but focused explicitly on grain micronutrient outcomes rather than yield alone, could provide the shared protocols, benchmark datasets, and model intercomparison needed to reduce uncertainty and produce more geographically resolved projections of climate-related nutritional risk (Rosenzweig et al., 2013).

### 5.7 Meta-analysis and data synthesis

Meta-analysis has played a central role in defining the current consensus on micronutrient dilution under eCO_2_ (Loladze, 2014). Through integrating results across species, environments, and experimental platforms, meta-analysis has helped quantify the average decline in grain Fe and Zn and identify major moderators such as water availability and nitrogen supply (Al-Hadeethi et al., 2019). These syntheses are especially valuable because individual studies often differ in cultivar, soil type, experimental duration, and reporting format, making direct comparison difficult. At the same time, meta-analysis is only as strong as the underlying evidence base. Heterogeneous reporting of mineral concentration, inconsistent separation of grain tissues, and unequal representation of FACE and chamber studies all remain important limitations (Dietterich et al., 2015). Future data synthesis would benefit from more standardized reporting, better metadata on soil and management conditions, and wider sharing of raw grain elemental data across experiments (Al-Hadeethi et al., 2019; Ainsworth and Long, 2021).

### 5.8 Knowledge gaps and future priorities

Despite substantial progress, several major questions remain unresolved. First, the relative importance of reduced transpiration-driven mass flow versus direct changes in transporter regulation remains unclear, and settling this will require experiments that combine tracer analysis with molecular profiling under realistic field conditions (Xue et al., 2015). Second, the responses of Se, I, Cu, B, and Mo remain far less certain than those of Fe and Zn because these species have rarely been studied systematically in multifactor field experiments (Mota et al., 2024). Third, it is still unclear whether biofortified genotypes that perform well under present conditions will retain their nutritional advantage under eCO2 combined with heat, drought, or nutrient limitation (Patil et al., 2025). Fourth, the long-term role of soil organic matter, microbial dynamics, and changing soil fertility under sustained eCO2 remains insufficiently resolved (Butterly et al., 2016). Finally, there is a pressing need to test whether molecular or breeding-based enhancement of nutrient uptake and grain loading can offset micronutrient losses under realistic future climates. These knowledge gaps define the next phase of research in this field: a shift from documenting the problem to identifying mechanisms that can be manipulated to boost nutritional resilience.

## 6 Implications and mitigation: safeguarding grain micronutrients in a high-CO_2_, warmer world

The evidence reviewed in the preceding sections shows that micronutrient dilution under eCO_2_ is not a minor or isolated physiological anomaly (Loladze, 2014). It is a recurring outcome of altered carbon gain, reduced transpiration-driven nutrient transfer, and changes in nutrient uptake and grain loading, and these effects may be intensified by drought, warming, and inadequate nitrogen supply (Al-Hadeethi et al., 2019). The practical implication is clear: protecting crop yield alone will not be sufficient. Strategies for future food security must also protect grain nutritional quality, especially Fe and Zn, which account for much of the public-health burden of hidden hunger (Myers et al., 2014). Successful mitigation will therefore require a layered approach which combines genetic improvement, agronomic management, soil and microbiome support, food-system interventions, and better decision tools (Bouis and Saltzman, 2017).

### 6.1 Breeding and genetic approaches

Genetic improvement remains the most durable mitigation pathway because it has the potential to maintain higher grain Fe and Zn concentrations without needing repeated external inputs every season. Biofortification programs in rice and wheat have already identified substantial genetic variation for grain Zn and Fe accumulation, which is being exploited through conventional breeding, marker-assisted selection, and transgenic or gene-based strategies (Wani et al., 2022). In rice, breeding and genomic approaches targeting grain Zn and Fe enhancement, including pathways related to nicotianamine synthesis and metal transport, have shown notable promise under greenhouse and conventional field conditions. In wheat, biofortification strategies combining genetic resources, physiological screening, and molecular tools have similarly demonstrated that grain Fe and Zn can be improved through breeding (Govindan et al., 2022). However, a key unresolved question is whether nutrient-dense genotypes retain their advantage under eCO_2_ combined with heat or drought. For this reason, the next essential step is to evaluate biofortified germplasm in multifactor field platforms that explicitly include future climatic conditions. Until such validation becomes routine, breeding should be viewed as highly promising, but not yet fully climate-proof (Senguttuvel et al., 2023; Velu et al., 2014).

An important question underlying all breeding-based mitigation is whether selection for high yield is inherently in tension with selection for grain nutrient density. The carbon–nutrient dilution mechanism described in Section 2.1 implies that, all else equal, genotypes bred primarily for larger grain or higher starch accumulation could see mineral concentration decline further simply because yield increases outpace mineral uptake and loading a tension that eCO_2_ is likely to sharpen rather than create.

Grain-filling duration is a further complicating factor: because warming shortens the grain-filling period (Section 4.1), cultivars selected for rapid grain filling under a warmer, high-CO_2_ climate may have even less time to load Fe and Zn into the grain, independent of any direct CO_2_ effect on transporter activity. Existing biofortification programmes demonstrate that yield and nutrient density are not mutually exclusive breeding targets, but achieving both together requires nutrient-density traits to be selected simultaneously with, rather than after, yield traits including explicit screening of candidate high-yield lines for grain Fe and Zn concentration under eCO_2_ and shortened grain-filling conditions before their release.

### 6.2 Global biofortification initiatives: the HarvestPlus experience

Beyond experimental breeding and agronomic interventions, large-scale international biofortification initiatives provide important evidence that crop-based approaches can translate micronutrient enhancement into meaningful nutritional gains. A prominent example is the HarvestPlus program, which has pioneered the development and dissemination of staple crop varieties enriched with iron, zinc, and provitamin A carotenoids, particularly across micronutrient-deficient populations in Africa and Asia. HarvestPlus and its partners have used conventional breeding, supported by genetic diversity screening and crop improvement approaches, to develop nutrient-dense varieties that combine enhanced micronutrient concentrations with agronomically desirable traits, farmer acceptability, and adaptation to local production systems (Bouis et al., 2019; Bouis and Saltzman, 2017). Biofortified crops developed through these efforts include iron-biofortified beans and pearl millet, zinc-biofortified wheat and rice, and provitamin A-biofortified maize, cassava, and orange-fleshed sweet potato. Evidence synthesized from HarvestPlus-supported interventions indicates that consumption of biofortified crops can improve micronutrient status, including iron status and vitamin A status, demonstrating the potential of crop improvement to address hidden hunger at population scale (Bouis and Saltzman, 2017; Huey et al., 2022). Importantly, the HarvestPlus experience demonstrates that biofortification is not limited to the development of nutrient-dense germplasm but requires coordinated efforts involving breeding, varietal release, seed systems, farmer adoption, value chains, consumer acceptance, and nutrition education. This integrated approach is particularly relevant under eCO_2_, where conventional nutrient-dense varieties may themselves experience micronutrient dilution. Thus, future biofortification programs should evaluate whether existing high-Fe and high-Zn germplasm can maintain their nutritional advantage under eCO_2_ and combined stresses such as drought, warming, and nitrogen limitation. Linking the established global biofortification infrastructure of initiatives such as HarvestPlus with climate-resilient breeding and multi-environment testing could therefore provide a practical pathway for safeguarding grain micronutrient density under future atmospheric CO_2_ conditions.

### 6.2 Agronomic biofortification

Among currently available interventions, agronomic biofortification is one of the most immediately deployable. Foliar application of Zn and Fe during reproductive stages has repeatedly been shown to increase grain micronutrient concentrations, often more effectively than soil application because it bypasses important constraints on root uptake and soil fixation (Velu et al., 2014). In wheat, recent field-scale work and broader reviews confirm that foliar application can substantially improve grain Zn and Fe concentration, although the response depends on genotype, timing, nutrient formulation, and environmental conditions (Ram et al., 2024). This makes foliar micronutrient management especially attractive as a near-term adaptation strategy in production systems where breeding gains are still being developed or where soil constraints restrict nutrient uptake efficiency. Its limitations are equally important: repeated application increases input cost, field response may be weaker under severe drought, and the benefits can vary across settings. Even so, agronomic biofortification remains one of the most practical short- to medium-term tools for partially offsetting the nutritional penalty of eCO2 (Velu et al., 2014; Ram et al., 2024).

### 6.3 Soil and water management

Soil and water management can also influence the extent to which eCO_2_ reduces grain micronutrient concentration, although the evidence here is more context-dependent than for breeding or foliar fertilization. Practices that improve soil organic matter, maintain aggregation, and support biologically active rhizospheres may help stabilize micronutrient availability and root function under climate stress (Kane et al., 2021). Likewise, management systems that reduce severe water stress during reproductive development can moderate the decline in transpiration-driven nutrient delivery to roots and grains (El-Hendawy et al., 2024). Conservation-oriented systems, residue retention, and organic amendments may therefore provide indirect support for micronutrient resilience by improving soil physical condition, buffering chemical extremes, and supporting root–microbe interactions (Rosinger et al., 2025). However, the current evidence is insufficient to claim a universal quantitative recovery effect across cropping systems. Soil and water management should thus be framed as an enabling component of mitigation rather than as a stand-alone solution.

### 6.4 Microbiome-based approaches

Microbiome-based mitigation is an emerging frontier rather than a mature field-ready strategy. A growing body of work suggests that arbuscular mycorrhizal fungi and beneficial rhizobacteria can enhance the acquisition of relatively immobile nutrients, including Zn and, sometimes, Fe, through mechanisms such as improved soil exploration, siderophore production, organic acid release, and altered rhizosphere chemistry (Watts-Williams and Cavagnaro, 2018). These findings make microbiome engineering an attractive concept in the context of eCO_2_, where root exudation patterns and plant–microbe interactions are likely to shift (Cotton et al., 2019). However, the present evidence under eCO_2_ remains limited, especially under realistic field conditions (Williams et al., 2018). Most available studies demonstrate potential rather than stable agronomic performance across environments. Accordingly, microbiome-based strategies should be regarded as promising complements to breeding and agronomy, but not yet as proven large-scale solutions for maintaining grain mineral quality under future climates.

### 6.5 Post-harvest and dietary strategies

Because production-side mitigation will not fully eliminate micronutrient decline in all systems, post-harvest and dietary strategies remain essential (Semba et al., 2022). Fortification of wheat flour and rice can help compensate for lower Fe and Zn concentrations in raw grains, especially among populations that rely heavily on refined cereal staples (Keats et al., 2019). At the same time, diversification of diets toward legumes, millets, and other nutrient-dense foods can reduce dependence on a narrow set of cereals that are especially vulnerable to eCO_2_ dilution (Satyavathi et al., 2021). This strategy is particularly relevant because C_4_ crops and many pulses often show a smaller nutritional penalty than C_3_ cereals under eCO_2_, or offer complementary nutrient profiles that improve overall dietary adequacy. Traditional varieties, landraces, and underutilized crops may also contribute meaningfully where local food systems still retain crop diversity. In practice, the strongest food-system response is likely to combine fortification, diversification, and targeted promotion of nutrient-dense staples rather than relying on any single dietary intervention.

### 6.6 Integrated modelling and decision support

Decision support will be increasingly important as the nutritional impacts of eCO_2_ become more spatially uneven. Crop–climate–nutrition models provide a means of identifying where grain micronutrient decline is most likely to overlap with cereal-dominant diets, poverty, and pre-existing Fe and Zn deficiency. Existing modelling studies already show that eCO_2_ can worsen human nutritional risk at large geographic scales, including projections of substantial increases in populations vulnerable to inadequate zinc intake. These models are not yet complete: they still represent root–soil interactions, grain mineral allocation, and less-studied elements such as Se, I, and Cu only imperfectly. Even so, they provide an important starting point for targeting investments in breeding, agronomic support, and public-health responses. The most useful next generation of decision tools will combine experimental mineral-response data with dietary surveys, soil information, and climate projections to identify regions where nutritional vulnerability is likely to intensify most rapidly (Smith and Myers, 2018; Beach et al., 2019).

### 6.7 Policy levers and institutional action

The mitigation challenge is not only technical; it is also institutional. Grain nutritional quality is rarely monitored with the same urgency as yield, creating a blind spot in climate adaptation planning. Integrating Fe and Zn concentration targets into crop improvement programs, procurement standards, fortification policy, and food security monitoring would help align agricultural and public health agendas (Ebi and Loladze, 2019). Public investment can accelerate this transition by supporting the deployment of nutrient-dense cultivars, implementing foliar micronutrient programs where appropriate, and strengthening surveillance of grain quality in national food systems (Bouis and Saltzman, 2017). Internationally, a more systematic approach to monitoring cereal micronutrient trends would improve early warning capacity and help identify where climate-mediated nutritional decline is becoming a major risk (Rembold et al., 2019). In this sense, policy action is less a separate mitigation strategy than the framework that makes all other strategies durable and scalable. Short-, medium-, and long-term policy actions for mitigating climate-driven micronutrient decline is given in **Table 4**.

**Table 4:** Short-, medium-, and long-term policy actions for mitigating climate-driven micronutrient decline.

Policy level	Specific action	Timeline
National	Establish routine monitoring of Fe and Zn concentrations in major staple grains and integrate nutritional-quality indicators into crop improvement and food-security surveillance. Integrating grain Fe/Zn thresholds into public procurement and school-feeding standards**. (**GAIN & HarvestPlus, 2020; FAO, 2026)	Short-term
Regional	Deploy targeted agronomic biofortification, including foliar Fe/Zn application where locally validated, alongside nutrient-dense cultivars. Regional seed-system harmonization (e.g., joint variety release/seed certification across regional economic blocs like ECOWAS or COMESA) alongside foliar programs, since this is often the actual bottleneck for biofortified variety uptake. (FAO, 2026; HarvestPlus)	Medium-term
Global	Integrate nutritional quality and climate-related micronutrient risks into NDCs and climate-adaptation frameworks (FAO, 2024; UNFCCC, 2025)	Long-term

### 6.8 Synthesis and priority actions

No single intervention will fully offset the micronutrient losses associated with eCO_2_ across all environments. The most realistic strategy is therefore a portfolio approach. In the near term, agronomic biofortification and food-system diversification offer the fastest deployable benefits (Velu et al., 2014). In the medium term, broader deployment of nutrient-dense germplasm can provide more durable improvement, especially when combined with supportive soil and nutrient management. In the longer term, the greatest gains are likely to come from integrating breeding, physiology, microbiome science, and predictive modelling into climate-ready crop improvement pipelines. Geographic prioritisation should focus on regions where cereal-dominant diets, drought risk, and micronutrient deficiency already overlap, because these areas are most likely to experience the sharpest nutritional consequences of rising atmospheric CO_2_. Overall, the central message is straightforward: safeguarding grain micronutrient quality in a warmer, high-CO_2_ world will require moving beyond yield-centred adaptation and treating nutritional resilience as a core goal of crop science and food policy (Semba et al., 2022).

### 6.9 Geographic vulnerability hotspots

The nutritional risk associated with eCO_2_-driven micronutrient dilution is not evenly distributed across low- and middle-income countries; it is shaped by the interaction between the CO_2_-sensitivity of a region’s dominant staple crop and the pre-existing burden of Fe and Zn deficiency in its population. South Asia represents one of the clearest hotspots: diets in this region are dominated by rice and wheat, both C3 cereals that show the most consistent grain Fe and Zn declines under eCO_2_ (Sections 3.1, 3.2), and the region already carries a high baseline prevalence of iron and zinc deficiency (Myers et al., 2015).

Sub-Saharan Africa presents a more heterogeneous picture. Large parts of the region depend on maize, sorghum, and millet, which are C4 crops and are therefore expected to show a smaller direct carbon-dilution penalty (Section 4.6); however, other areas including major wheat- and rice-consuming populations in East Africa remain exposed to the same C3 dilution mechanisms as South Asia, and the region as a whole faces compounding risk from drought and heat stress that can independently reduce yield and grain quality regardless of photosynthetic pathway (Chukwudi et al., 2021).

This distinction matters for policy: interventions premised on a uniform ‘LMIC’ (Low- and Middle-Income Country) nutritional threat risk misallocating resources, whereas differentiating hotspots by staple-crop photosynthetic pathway, dietary dependence, and existing deficiency prevalence would allow breeding and biofortification investment to be targeted where the CO_2_-related penalty is mechanistically largest (South Asian rice and wheat systems) and where climate-stress-driven yield and quality losses are the dominant concern (Sub-Saharan African maize- and sorghum-based systems). Overlaying grain-mineral response data with dietary composition and deficiency-prevalence maps, as called for in Section 6.6, would allow this hotspot logic to be made spatially explicit in future assessments for example as a simple two-axis figure plotting staple-crop C3/C4 exposure against baseline Fe/Zn deficiency prevalence by region.

## 7 Conclusion

Rising atmospheric CO_2_ has often been discussed mainly in terms of its potential to stimulate photosynthesis and increase crop productivity. This review shows that such a view is incomplete. Across major staple crops, eCO_2_ also reduces grain micronutrient concentration, especially for Fe and Zn, through a combination of interacting processes: reduced transpiration-driven nutrient delivery, imbalance between carbon accumulation and mineral acquisition, and changes in uptake, transport, and grain loading. Evidence from FACE experiments, controlled-environment studies, systematic reviews, and meta-analyses now makes it clear that this decline is not an isolated or occasional response, but a recurring feature of crop growth under eCO_2_.

The nutritional implications are substantial. In wheat, rice, and several legumes, grain Fe and Zn commonly decline under eCO_2_, and these declines can become more severe when CO_2_ enrichment is combined with drought, warming, or low nitrogen supply. This is especially concerning because the populations most vulnerable to hidden hunger are also those most dependent on cereal-based diets for mineral intake. Yield gains under eCO_2_ do not compensate for reduced grain nutrient density when diet quality is already marginal. In that sense, the challenge posed by eCO_2_ is not only about food production but also about food quality and nutritional adequacy.

At the same time, the response is not uniform across elements. Fe and Zn show the clearest and most consistent declines, whereas responses of Cu and Mn are more variable, and evidence for Se, I, B, and Mo remains limited. This unevenness highlights two important conclusions. First, hidden-hunger assessments focused only on Fe and Zn may still underestimate the broader nutritional implications of rising CO_2_. Second, there remains a strong need for multi-element studies under realistic field conditions, especially for micronutrients that are important to human health but poorly represented in current crop-climate datasets.

A major strength of the field now is that the problem is no longer only descriptive. Mechanistic understanding has advanced enough to identify realistic intervention points. Breeding and biofortification can improve the baseline mineral density of grain; agronomic biofortification offers an immediately deployable strategy; soil and water management can help stabilise nutrient acquisition under stress; and dietary diversification and fortification can buffer the public-health consequences where production-side solutions alone are insufficient. No single intervention will be universally effective, but the evidence supports a portfolio approach that combines genetic, agronomic, ecological, and food-system responses.

The central message of this review is therefore both cautionary and constructive. eCO_2_ is likely to intensify micronutrient insecurity unless grain nutritional quality is treated as a core target of climate adaptation. Yet the same body of evidence that defines the problem also points toward workable solutions. What is needed now is stronger integration across plant physiology, breeding, agronomy, nutrition, and policy. Future crop improvement programs must evaluate nutritional resilience alongside yield and stress tolerance, and future food-security planning must account for the possibility that crops can become more abundant while becoming less nourishing. eCO_2_ may increase the quantity of food produced, but unless nutritional quality is protected, it will also erode one of the most essential functions of agriculture: supplying the minerals required for human health.
